# Proton channel HVCN1 is required for effector functions of mouse eosinophils

**DOI:** 10.1186/1471-2172-14-24

**Published:** 2013-05-24

**Authors:** Xiang Zhu, Eucabeth Mose, Nives Zimmermann

**Affiliations:** 1 Division of Allergy and Immunology, Department of Pediatrics, Cincinnati Children’s Hospital Medical Center and the University of Cincinnati College of Medicine, Cincinnati, OH 45229, USA

**Keywords:** Proton channel, HVCN1, Eosinophil

## Abstract

**Background:**

Proton currents are required for optimal respiratory burst in phagocytes. Recently, HVCN1 was identified as the molecule required for the voltage-gated proton channel activity associated with the respiratory burst in neutrophils. Although there are similarities between eosinophils and neutrophils regarding their mechanism for respiratory burst, the role of proton channels in eosinophil functions has not been fully understood.

**Results:**

In the present study, we first identified the expression of the proton channel HVCN1 in mouse eosinophils. Furthermore, using HVCN1-deficient eosinophils, we demonstrated important cell-specific effector functions for HVCN1. Similar to HVCN1-deficient neutrophils, HVCN1-deficient eosinophils produced significantly less reactive oxygen species (ROS) upon phorbol myristate acetate (PMA) stimulation compared with WT eosinophils. In contrast to HVCN1-deficient neutrophils, HVCN1-deficient eosinophils did not show impaired calcium mobilization or migration ability compared with wild-type (WT) cells. Uniquely, HVCN1-deficient eosinophils underwent significantly increased cell death induced by PMA stimulation compared with WT eosinophils. The increased cell death was dependent on NADPH oxidase activation, and correlated with the failure of HVCN1-deficient cells to maintain membrane polarization and intracellular pH in the physiological range upon activation.

**Conclusions:**

Eosinophils require proton channel HVCN1 for optimal ROS generation and prevention of activation-induced cell death.

## Background

Eosinophils are bone marrow (BM)-derived granulocytes that are implicated in of numerous inflammatory responses, especially allergic diseases and parasitic helminth infections [1]. In response to diverse stimuli, eosinophils are recruited from the circulation to the inflammatory foci, where they secrete an array of pro-inflammatory factors upon activation, including chemokines, cytokines and reactive oxygen species (ROS). Thus, understanding the mechanisms regulating eosinophil effector functions may reveal important implications for eosinophilic inflammatory responses.

ROS generation in eosinophils is dependent on assembly and activation of the normally latent NADPH oxidase complex, which is located in the plasma membrane and has strong similarities to the NADPH oxidase complex in neutrophils [2,3]. Upon activation, the NADPH oxidase transports electrons across the membrane to reduce molecular oxygen, O_2_, into superoxide, O_2_^-^, which undergoes non-enzymatic or superoxide dismutase (SOD)-catalyzed dismutation to hydrogen peroxide, H_2_O_2_. In turn, hydrogen peroxide can be used by eosinophil peroxidase or neutrophil myeloperoxidase to form HOBr or HOCl, respectively [4]. During this process, the NADPH oxidase is electrogenic and voltage dependent [5-7], and its sustained production of superoxide requires the movement of a compensating charge across the membrane, i.e. efflux of positively charged ions or influx of negatively charged ions. Multiple studies have found that most of the compensating charge is carried by protons that are released in the cytosol by the conversion of NADPH to NADP^+^ and H^+^ and subsequently efflux via voltage-gated proton channels [7]. This efflux of protons simultaneously provides the compensating charge and prevents the acidification of the cytosol. In 2006, the voltage-gated proton channel was molecularly identified in humans and mice [8,9]. More recently, the generation of viable mice bearing a disrupting mutation within the *Hvcn1* gene encoding the voltage-gated proton channel prompted a wave of new studies on proton channel function and regulation [10,11]. A few studies found that phorbol myristate acetate (PMA)-activated HVCN1-deficient neutrophils produced less ROS than neutrophils from WT mice [10,12], which suggests that HVCN1 is required for high-level NADPH oxidase-dependent superoxide production during phagocyte respiratory burst. Unexpectedly, loss of HVCN1 also decreased neutrophil migration ability *in vitro*[12]. Moreover, HVCN1 deficiency in B cells downregulated B cell receptor signaling and antibody responses *in vivo*[13]. Taken together, these findings identified unanticipated and varied functions for HVCN1 in neutrophils and B cells and underscored the need to study individual cell types.

In human eosinophils, voltage-gated proton channel activity has been shown by detection of proton current during ROS production [14,15]. Recently, it was documented that human eosinophils express HVCN1 [16]. However, although there are similarities between eosinophils and neutrophils in regard to NADPH oxidase-mediated ROS production, the location of NADPH oxidase and the amount of ROS production mediated by NADPH oxidase largely vary in these two cell types. The majority of eosinophil NADPH oxidase is located on the plasma membrane; thus, eosinophils primarily generate extracellular ROS to kill nonphagocytosable targets. In contrast, the vast majority of neutrophil NADPH oxidase is located on the phagosome; thus, neutrophils primarily generate intracellular ROS to kill invading microorganisms [17]. Additionally, eosinophils express more NADPH oxidase than neutrophils, thus generating 3–6 times more superoxide than neutrophils upon activation [18-20].

In the present study, we examined whether HVCN1 is expressed by mouse eosinophils and required for ROS generation, eosinophil chemotaxis or other functions. First, we identified that *Hvcn1* mRNA is expressed at a higher level in allergic lung and in mouse eosinophils compared with neutrophils. Second, we determined that, unlike in neutrophils, HVCN1 deficiency does not affect calcium flux or migration of mouse eosinophil. Finally, in the absence of HVCN1, eosinophils undergo significantly increased cell death upon PMA stimulation which is dependent on NADPH oxidase activity. This increased activation-induced cell death is likely caused by membrane depolarization and cytosolic acidification in HVCN1-deficient eosinophils following PMA stimulation. Collectively, our data demonstrate that HVCN1 is important in regulating eosinophil effector functions.

## Methods

### Mice

Mice bearing a targeted disruption in the HVCN1 gene (*Hvcn1*^−/−^, backcrossed eleven times onto the C57BL/6 background) were previously described [10]. In early experiments, we used WT C57BL/6 mice as a control. In later experiments, we used heterozygous-derived WT and *Hvcn1*^−/−^ lines (backcrossed twelve times onto the C57BL/6 background). For each outcome (cell death, ROS production, intracellular pH, membrane depolarization), the difference between WT and *Hvcn1*^−/−^ eosinophils was similar irrespective of the type of control used. Mice used for the studies were between 5 and 10 weeks old. All mice were housed under specific pathogen-free conditions and treated in accordance with institutional guidelines. Studies were approved by the Cincinnati Children’s Hospital Medical Center IACUC (protocol number 0D11085). Pain and suffering were alleviated by anesthesia during procedures and mice were sacrificed by CO_2_ inhalation.

### Mouse model of allergic airway inflammation

Mice were challenged intranasally with *Aspergillus fumigatus* as described [21]. Briefly, 100 μg (50 μl) of *A. fumigatus* extract or 50 μl of normal saline solution alone was applied to the mouse nasal cavity 3 times per week for 3 weeks. 18 hours after the last challenge, mice were sacrificed by CO_2_ inhalation. Bronchoalveolar lavage fluid (BALF) was collected and infiltrating cells differentiated as previously described [22]. To isolate BALF eosinophils, the infiltrating cells were adhered in a 6-well plate at 37°C and 5% CO_2_ incubator. One hour later, the nonadherent fraction containing ~85% purified eosinophils was recovered. These BALF eosinophils and whole lung tissue were used for total RNA extraction.

### Microarray analysis

Microarray data for *Hvcn1* expression are from a previously published data set [23]. Briefly, the genome-wide mouse MOE430 2.0 GeneChip (Affymetrix, Santa Clara, CA) was used. Average difference used in the present study is a quantitative measure of the level of gene expression, calculated by taking the difference between mismatch and perfect match of every probe pair and averaging the differences over the entire probe set [21].

### Eosinophil counting and culture

Peripheral blood eosinophils were counted by Discombe’s staining [24]. BM-derived eosinophils were produced according to the method described in reference [25] with minor modifications. Briefly, BM progenitor cells were collected from the femurs and tibiae by flushing the opened bones with IMDM medium (Invitrogen). A hypotonic lysis was performed to eliminate red blood cells. Then the cells were cultured in six-well plates at 1×10^6^/ml in IMDM containing 10% FBS (Cambrex), 100 IU/ml penicillin and 10 μg/ml streptomycin (Cellgro), 2 mM glutamine (Invitrogen), and 50 μM 2-Mercaptoethanol (Sigma-Aldrich) supplemented with 100 ng/ml stem cell factor (SCF; PeproTech) and 100 ng/ml FLT3 ligand (PeproTech) from days 0 to 4. On day 4, the medium was replaced with fresh medium containing 10 ng/ml recombinant mouse IL-5 (R&D systems). From this point forward, half of the medium was replaced every other day with fresh medium containing IL-5, and the cell density was maintained around 1×10^6^/ml. On day 14, the cells were harvested for *in vitro* experiments after flow cytometric identification by CCR3-FITC (R&D Systems) and Siglec-F-PE (BD Bioscience) staining as well as morphological examination by cytospun slide staining with a modified Giemsa preparation (Diff Quik). As expected, more than 90% of harvested cells are eosinophils (data not shown).

### Neutrophil culture

BM-derived neutrophils were produced according to the protocol described in [26] with minor modifications. Briefly, BM progenitor cells were cultured in the IMDM medium (Invitrogen) containing 10% FBS (Cambrex), 100 IU/ml penicillin and 10 μg/ml streptomycin (Cellgro), 2 mM glutamine (Invitrogen), and 50 μM 2-Mercaptoethanol (Sigma-Aldrich) supplemented with 100 ng/ml SCF (PeproTech) and 50 ng/ml granulocyte-colony stimulating factor (PeproTech) from day 0 to 6. After six days, SCF was withdrawn to arrest proliferation and induce further differentiation. On day 8, the cells were harvested followed by flow cytometric identification by staining with Gr-1-APC (eBioscience). The morphology of cytospun cells was determined by staining with a modified Giemsa preparation (Diff Quik).

### Isolation of neutrophils and differential counting in peritonitis model

4% thioglycollate medium was intraperitoneally injected into WT and HVCN1-deficient mice to induce peritonitis as described [27]. To isolate and count neutrophils, the peritoneal lavage cells were collected 4 hours after injection and adhered in a 6-well plate at 37°C and 5% CO_2_ incubator. One hour later, the nonadherent fraction containing 99% purified neutrophils was recovered and counted. To count the numbers of infiltrating eosinophils in the peritonitis model, we collected peritoneal lavage cells 48 hours after thioglycollate injection and performed differential counting on Diff Quik-stained cytospin slides.

### Total RNA extraction and real-time RT-PCR

Total RNA from BM-derived eosinophils and neutrophils, BALF eosinophils, neutrophils isolated from the peritonitis model, and lung tissue from saline- or *A. fumigatus*-challenged mice was extracted by TRIzol (Invitrogen) as per the manufacturer’s instructions. One microgram of RNA was subjected to DNase I treatment (Qiagen) and reverse transcribed using the iScript™ cDNA Synthesis Kit (BioRad). Two microliters of cDNA were subjected to real-time RT-PCR set up with iQ™ SYBR Green Supermix (BioRad) and primer sets for mouse *Hvcn1* (forward: 5′-TCGTGCTTGCTGAACTCCTCCT; reverse: 5′-GGCAAAGCTCATGTAGTGGAACG) and *Actb* (β-actin gene) (forward: 5′-CGATGCCCTGAGGCTCTTTTCC; reverse: 5′-CATCCTGTCAGCAATGCCTGGG) separately. The relative expression levels of *Hvcn1* were normalized to the housekeeping gene *Actb*.

### Western blotting

In brief, protein lysates from mature WT and HVCN1-deficient BM-derived eosinophils were prepared by Laemmli sample buffer (BioRad) with protease inhibitor cocktail (Roche) and sonication (4 rounds of 10 seconds at 50% power). After blocking with 5% non-fat milk, the nitrocellulose membrane was probed with the primary rabbit polyclonal anti-HVCN1 (1:1000 dilution; AbCam) overnight at 4°C and the secondary antibody (horseradish peroxidase [HRP] conjugated-anti-rabbit, 1:1000 dilution; Cell Signaling) for 1 hour at room temperature. Signals were detected on X-ray films using the enhanced chemiluminescence method (ECL™ Western Blotting Analysis System; GE Healthcare). Similar results were obtained with rabbit polyclonal anti-HVCN1 (4234; kind gift from Dr. I. Scott Ramsey).

### Measurement of ROS production

For H_2_O_2_ measurements, mature BM-derived eosinophils were suspended in HBSS (Invitrogen) containing 25 μM Amplex Red (Invitrogen) and 0.05 U/ml HRP and then seeded in a 96-well flat-bottom plate with 20,000 cells per well in a 100-μl final volume. After pre-incubation for 10 minutes at 37°C, the inhibitors Zn^2+^ and diphenylene iodonium (DPI; Sigma) were added at the indicated concentrations 10 minutes before the recording. PMA (50 ng/ml) was added at time t = 0, and fluorescence was measured at 590 nm on a microplate reader (BioTek Synergy 2) every 10 minutes for 1 hour at 37°C. Because phosphate in HBSS may chelate Zn^2+^, in some experiments we tested phosphate-free Ringer’s solution (160mM NaCl, 5mM KCl, 2mM CaCl_2_, 1mM MgCl_2_, 5mM HEPES and 10mM glucose) in parallel with HBSS [28]. Alternatively, to measure superoxide production, we pre-incubated cells with 200 μM lucigenin (Invitrogen) in HBSS for 30 minutes at 37°C in the presence or absence of SOD (1000 U/ml); using the same microplate reader, the chemiluminescence was measured every 10 minutes following the addition of PMA (50 ng/ml). For both assays, each condition was performed in triplicate. To measure the intracellular ROS, cells were pre-incubated with 1 μM dihydrorhodamine 123 (DHR 123; Invitrogen) for 30 minutes at 37°C. After subsequent PMA (50 ng/ml) stimulation in the presence or absence of Zn^2+^ and DPI, cells were subjected to flow cytometric analysis on FACS Canto II (BD Bioscience) for analysis.

### Membrane potential measurement

WT and *Hvcn1*^−/−^ eosinophil plasma membrane potential was determined using bis-(1,3-dibutylbarbituric acid)trimethine oxonol (DiBAC4(3); Invitrogen), a potential-sensitive bisoxonol fluorescent dye that enters depolarized cells and exhibits enhanced fluorescence (Invitrogen). Cells at 0.5×10^6^/ml were pre-incubated with 600 nM DiBAC4(3) in PBS at room temperature in the dark for 30 minutes. PMA (50 ng/ml) was added into the cell suspension at time t = 0 and fluorescence was recorded continuously by flow cytometry on Canto II (BD Bioscience) for 12 minutes with excitation at 488 nm and emission at 530 ± 30 nm.

### Intracellular pH measurement

Intracellular pH was measured as described previously [29] with minor modifications. Briefly, after stimulation with PMA (50 ng/ml), BM-derived eosinophils were suspended at 1×10^6^ cells/ml in PBS and loaded with 5 μM SNARF-4 AM (Invitrogen) for 30 minutes at 37°C in the dark. After incubation, eosinophils were centrifuged and resuspended in PBS. Emission fluorescence data were collected by flow cytometer Canto II (BD Bioscience) at 580 nm and 640 nm using linear amplification, and the fluorescence ratio 640 nm / 580 nm was determined after gating out dead cells that failed to retain SNARF-4 AM fluorescence. For calibration, 1 μg/ml nigericin was added to the unstimulated cell suspension containing 140 mM KCl, 1 mM MgCl_2_, 2 mM CaCl_2_, 5 mM α-D-glucose and 20 mM MES or Tris (pH 6.0-8.0). Intracellular pH of the test samples was calculated according to the curve developed from the calibration solutions.

### Assessment of cell viability

To determine the viability of BM-derived eosinophils after PMA (50 ng/ml) stimulation, cells were harvested and stained with 0.4% trypan blue solution at room temperature for 3 minutes. The live (unstained) and dead (stained) cells were then counted with a hemocytometer under a light microscope. Alternatively, the viability dye 7-AAD and APC-conjugated Annexin-V (BD Bioscience) were utilized to stain the eosinophils resuspended in Annexin-V binding buffer (BD Bioscience). Samples were incubated at room temperature for 15 minutes and analyzed immediately on flow cytometer Calibur I or Canto II (BD Bioscience).

### Chemotaxis assay

The assay was performed in a transwell plate (Corning) with a 5.0-μm pore size polycarbonate membrane. Five hundred microliters of PBS containing recombinant mouse Eotaxin-1 (mEotaxin-1 at 0, 1, 10, and 100 ng/ml, PeproTech) were placed in the lower chamber. 1×10^6^ BM-derived eosinophils in 100 microliters of PBS were placed in the upper chamber. PBS in lower and upper chamber was supplemented with 0.1% BSA and 1.0 mM CaCl_2_. After 4-hour incubation at 37°C, cells migrating to the lower chamber were harvested and counted in a hemocytometer.

### Intracellular calcium measurement

Eosinophils were loaded with 1 μM Fluo-4 AM (Invitrogen), a calcium-sensitive fluorescent dye, in the assay solution (HBSS containing 0.1% BSA and 1 mM CaCl_2_) and incubated in the dark for 1 hour at 37°C. Cells were washed twice and resuspended in the assay solution at the density of 1×10^6^ cells/ml. The fluorescence was recorded by flow cytometry on Canto II (BD Bioscience) with excitation at 488 nm and emission at 520 nm prior to and immediately after adding mEotaxin-1 (1, 10, 100, and 300 ng/ml).

### Statistical analysis

Student’s *t*-test and ANOVA were used for two-group and multiple-group statistical analysis, respectively. The software GraphPad Prism 5 was used for the analyses.

## Results

### Mouse eosinophils express HVCN1

**Figure 1:**
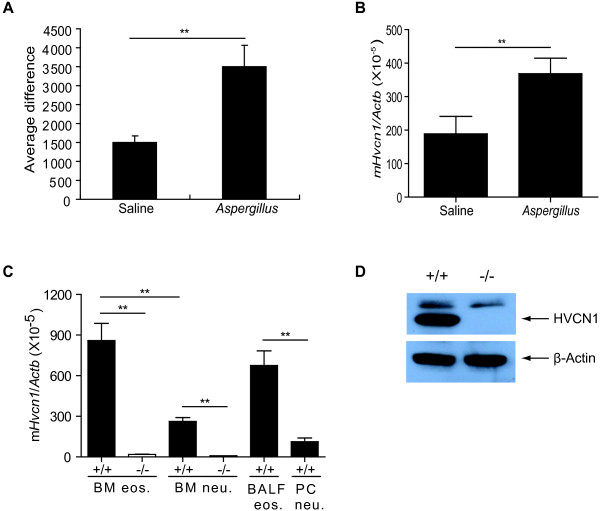
**HVCN1 mRNA and protein expression levels in lung tissue and eosinophils. **(**A-B**) The microarray (**A**) and real-time RT-PCR (**B**) analyses show increased *Hvcn1 *levels in the lung tissue of the allergen (*Aspergillus*)-challenged mice compared to saline-challenged controls. (**C**) Real-time RT-PCR analysis of *Hvcn1 *mRNA expression levels relative to housekeeping gene *Actb* (β-actin) in WT and HVCN1-deficient eosinophils and neutrophils from the different source (seen in “Methods”). Data are expressed as mean ± SD of 4–6 mice per group. **, *P *< 0.01. (**D**) Western blotting of HVCN1 in BM-derived eosinophils from WT and HVCN1-deficient mice, the representative of 4 experiments.

Previous microarray analysis [23] identified significantly increased lung expression of *Hvcn1* in allergen (*Aspergillus*)-challenged mice compared with saline-challenged mice (Figure 1A). This increase was further confirmed by real-time RT-PCR (Figure 1B) and could be explained by the recruitment of HVCN1-expressing cells or by the increased transcription of the *Hvcn1* in structural or recruited cells. As human eosinophils were recently reported to express HVCN1 at the mRNA and protein levels [16], we hypothesized that mouse eosinophils, as the major inflammatory cells in the allergic lung, also express HVCN1. By using real-time RT-PCR, we determined that *Hvcn1* is indeed expressed in mouse eosinophils not only derived from BM progenitor cells but also isolated from the lung tissue of allergen-challenged WT mice (Figure 1C). Furthermore, we observed that *Hvcn1* mRNA expression level in BM-derived eosinophils is significantly higher (3.3-fold on average) than that in BM-derived neutrophils. Consistently, in BALF eosinophils *Hvcn1* expression level is also significantly higher (6.0-fold on average) than that in neutrophils isolated from a peritonitis model (Figure 1C). As a control, our data show that HVCN1 mRNA and protein expression level is markedly reduced in HVCN1-deficient eosinophils compared to that in WT eosinophils (Figure 1C and 1D). In order to explore the function of HVCN1, BM-derived eosinophils from WT and HVCN1-deficient mice were cultured *in vitro*. We found no significant difference in the development of BM-derived eosinophils from WT and HVCN1-deficient mice with regard to their total cell numbers, morphology, or co-expression of CCR3 and Siglec-F on the cell surface (data not shown). Consistent with these findings, we observed no significant difference in the number of peripheral blood eosinophils between WT and HVCN1-deficient mice (data not shown), suggesting that HVCN1 deficiency has no effect on eosinophil development *in vivo*.

### HVCN1 is required for optimal ROS production by eosinophils in response to PMA stimulation

**Figure 2:**
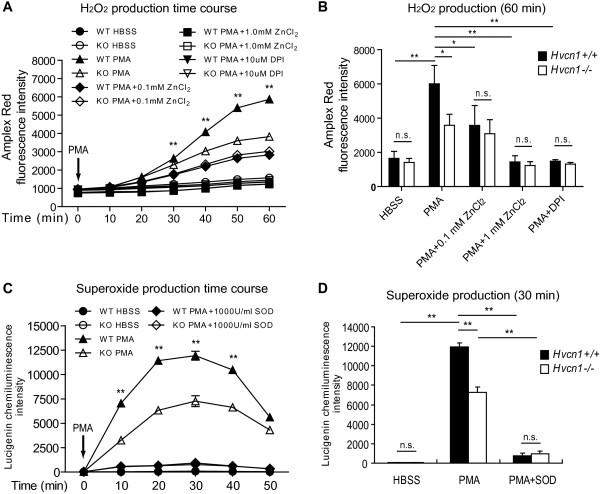
**ROS production from WT and HVCN1-deficient eosinophils. **(**A**) Time-dependent H_2_O_2 _production by WT and HVCN1-deficient (KO) BM-derived eosinophils was measured using HBSS solution containing 25 μM Amplex Red and 0.05 U/ml HRP. Eosinophils were stimulated by PMA (50 ng/ml) in the presence or absence of Zn^2+ ^or DPI at the indicated concentrations. Data are representative of 4 separate experiments and expressed as mean ± SD. The arrow indicates the addition of PMA at Time = 0. (**B**) Mean H_2_O_2 _production under the same conditions as (**A**) at 60 minutes following PMA stimulation. Data are expressed as the mean ± SD of 4 separate experiments done in triplicate. (**C**) Time-dependent superoxide production by WT and HVCN1-deficient BM-derived eosinophils was determined using HBSS solution containing lucigenin (200 μM). Eosinophils were stimulated by PMA (50 ng/ml) in the presence or absence of SOD (1000 U/ml). Data are representative of 4 separate experiments and expressed as mean ± SD. The arrow indicates the addition of PMA at Time = 0. (**D**) Mean superoxide production under the same conditions as (**C**) at 30 minutes after PMA stimulation. Data are expressed as the mean ± SD of 4 separate experiments done in triplicate. *, *P* < 0.05; **, *P *< 0.01; n.s., not significant.

We measured ROS production by WT and HVCN1-deficient eosinophils following PMA stimulation. As shown in Figure 2A and 2B, H_2_O_2_ production was reduced by 51% ± 3% (n = 4 experiments; *P* < 0.05) in HVCN1-deficient eosinophils compared with WT eosinophils following PMA stimulation, suggesting that HVCN1 is required in part for ROS production by activated eosinophils. Consistent with a previous report [28], the proton channel inhibitor Zn^2+^ reduced H_2_O_2_ production in a dose-dependent manner with complete inhibition at the concentration of 1 mM. Moreover, DPI, an inhibitor of flavoproteins including NADPH oxidase, also completely inhibited H_2_O_2_ production in both WT and HVCN1-deficient eosinophils (Figure 2A and 2B). As a control to avoid the formation of zinc phosphate in the H_2_O_2_ assay, phosphate-free Ringer’s solution substituted HBSS in the parallel experiments and similar results were obtained (data not shown). When ROS was measured with an alternative method utilizing the superoxide-specific probe lucigenin, we observed that HVCN1-deficient eosinophils had significantly reduced superoxide production compared to WT eosinophils as early as 10 minutes after PMA stimulation (Figure 2C) and that the average inhibition was 43 ± 4% (n = 4 experiments; *P* < 0.01, Figure 2D). To test the specificity of lucigenin, we used SOD in the assay. The addition of SOD quenched the chemiluminescence induced by both activated WT and HVCN1-deficient eosinophils to the baseline level. Similar to BM-derived eosinophils, bronchoalveolar lavage eosinophils from allergen-challenged mice showed decreased ROS accumulation in HVCN1-deficient cells (data not shown). Taken together, these results indicate that HVCN1 is required for optimal ROS release from eosinophils following PMA stimulation.

### HVCN1 deficiency does not affect eosinophil migration in vitro and in vivo

**Figure 3:**
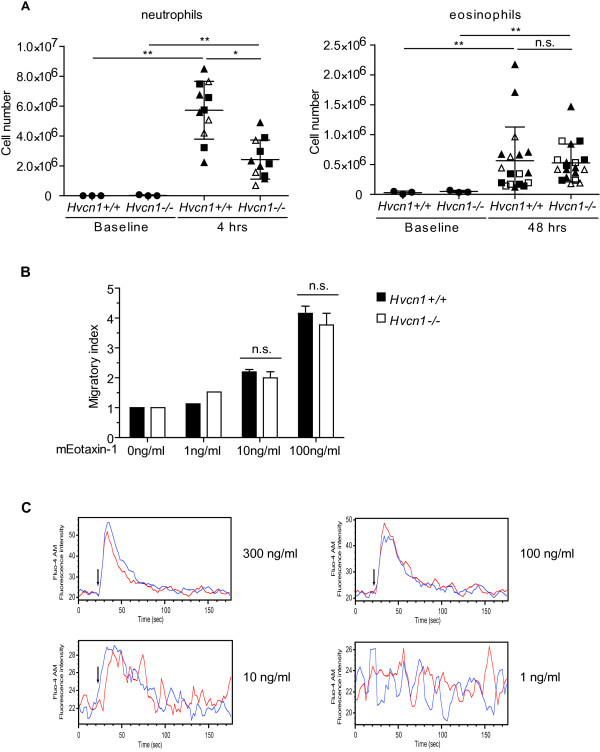
**Chemotaxis and intracellular calcium flux in WT and HVCN1-deficient eosinophils. **(**A**) The *in vivo *migration of neutrophils (left; n = 3 experiments) and eosinophils (right; n = 4 experiments) was determined by morphologically counting the cells collected from the peritoneal cavity of WT and HVCN1-deficient mice at the indicated time points following intraperitoneal injection of thioglycollate medium. Data are expressed as the mean ± SD with mice from each experiment indicated by different symbols. (**B**) Transwell migration assay was performed to compare the *in vitro *migration of WT and HVCN1-deficient eosinophils subjected to mEotaxin-1 at the indicated concentrations. The results are expressed as migratory index by determining the ratio of total cell number under mEotaxin-1 attraction to total cell number without mEotaxin-1. Data are representative of 3 experiments and expressed as mean ± SD. (**C**) Cytosolic Ca^2+ ^response in WT and HVCN1-deficient eosinophils was determined with intracellular fluorescence dye Fluo-4 AM in the presence of 1 mM extracellular Ca^2+^. Shown are the representative overlaid traces out of 5 experiments for mEotaxin-1 (1-300ng/ml). The arrow indicates the time of mEotaxin-1 addition. The trace (blue: WT eosinophils; red: HVCN1-deficient eosinophils) represents the average fluorescence intensity from cultured BM-derived eosinophils at the collection speed of approximately 150 cells per second by flow cytometer Canto II. *, *P* < 0.05; **, *P *< 0.01; n.s., not significant.

A previous study demonstrated that HVCN1-deficient neutrophils have defective migration in response to the chemoattractant fMIVIL *in vitro* secondary to impaired calcium responses in HVCN1-deficient neutrophils [12]. We now extend these findings *in vivo* in a model of peritonitis. Four hours after intraperitoneal injection of 4% thioglycollate medium, the infiltrating neutrophils in the peritoneal cavity were collected and counted. The number of infiltrating neutrophils in HVCN1-deficient mice was significantly reduced by 55% compared to that of WT mice (Figure 3A, left), suggesting that HVCN1 deficiency also impairs neutrophil migration *in vivo*. In contrast, no significant difference was observed in the number of recruited eosinophils between WT and HVCN1-deficient mice 48 hours post injection (Figure 3A, right), suggesting that HVCN1 deficiency does not affect eosinophil migration *in vivo*. Furthermore, we did not observe any difference in the *in vitro* chemotactic migration of WT and HVCN1-deficient eosinophils towards the eosinophil-selective chemoattractant eotaxin-1 (Figure 3B). Notably, WT and HVCN1-deficient eosinophils have similar viability following eotaxin-1 stimulation as determined by trypan blue staining (data not shown). Since the previous study had suggested that lack of HVCN1 impedes the migration of neutrophils by aborting physiological Ca^2+^ flux [12], we assessed Ca^2+^ flux in eosinophils upon the addition of mEotaxin-1. As shown in Figure 3C, we found that there was comparable intracellular calcium accumulation between WT and HVCN1-deficient eosinophils in response to mEotaxin-1 (*P*=0.79 by paired *t*-test, n=5 experiments). Collectively, these results suggest that HVCN1 deficiency does not affect mouse eosinophil migration *in vitro* and *in vivo*.

### Enhanced cell death in HVCN1-deficient eosinophils following PMA stimulation

**Figure 4:**
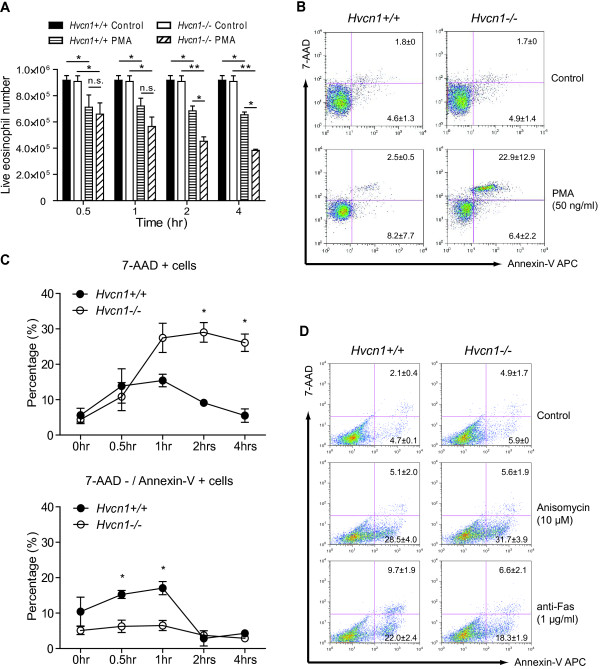
**Enhanced cell death in HVCN1-deficient eosinophils following PMA stimulation. **(**A**) The viable eosinophils at the indicated time points following PMA (50 ng/ml) stimulation were stained with trypan blue and counted. Data are expressed as the mean ± SD of 3 separate experiments performed in duplicate. (**B**) Analysis of eosinophil apoptosis / cell death after four-hour PMA (50 ng/ml) stimulation by flow cytometer Calibur I. Data are expressed as the mean ± SD of 4 separate experiments. (**C**) Kinetic analysis of eosinophil apoptosis / cell death following PMA (50 ng/ml) stimulation. (**D**)Analysis of eosinophil apoptosis / cell death after four-hour anisomycin (10 μM) or anti-Fas antibody (1 μg/ml) stimulation by flow cytometer Canto II. Data are expressed as the mean ± SD of 3 separate experiments. *, *P *< 0.05; **, *P *< 0.01; n.s., not significant.

During the course of our studies, we noticed differences in viability of wild type and HVCN1-deficient cells. Thus, we formally tested the hypothesis that HVCN1 deficiency affects eosinophil viability. PMA-stimulated HVCN1-deficient eosinophils underwent significantly increased cell death compared with PMA-stimulated WT eosinophils (Figure 4A). This significant decrease in viable PMA-treated HVCN1-deficient cells compared to viable PMA-treated WT cells was seen by 2 hours after PMA stimulation; the difference increased even further by 4 hours after stimulation, indicating that the number of eosinophils undergoing cell death is increased and that the kinetics of cell death are hastened for HVCN1-deficient cells. We used flow cytometry with 7-AAD and Annexin-V-APC staining to better understand the cell death mechanisms. As shown in Figure 4B, PMA-stimulated, HVCN1-deficient eosinophils were Annexin V-positive and 7AAD-positive at 4 hours after PMA stimulation. This finding was present at earlier time points (1–2 hours, Figure 4C), suggesting that PMA-stimulated, HVCN1-deficient eosinophils do not undergo characteristic apoptotic cell death. In order to test the possibility that HVCN1-deficient eosinophils are susceptible to an increased rate of cell death irrespective of the stimulus, we used several independent approaches to induce cell death in WT and HVCN1-deficient eosinophils. There was no difference between the two genotypes in cell viability in unstimulated eosinophils undergoing spontaneous apoptosis or in eosinophils induced to undergo apoptosis by IL-5 withdrawal or treatment with camptothecin, anisomycin or anti-Fas antibodies (data not shown and Figure 4D). Taken together, these data suggest that eosinophils lacking HVCN1 undergo enhanced cell death upon activation with PMA.

### Increased membrane depolarization and cytosolic acidification in activated HVCN1-deficient eosinophils

**Figure 5:**
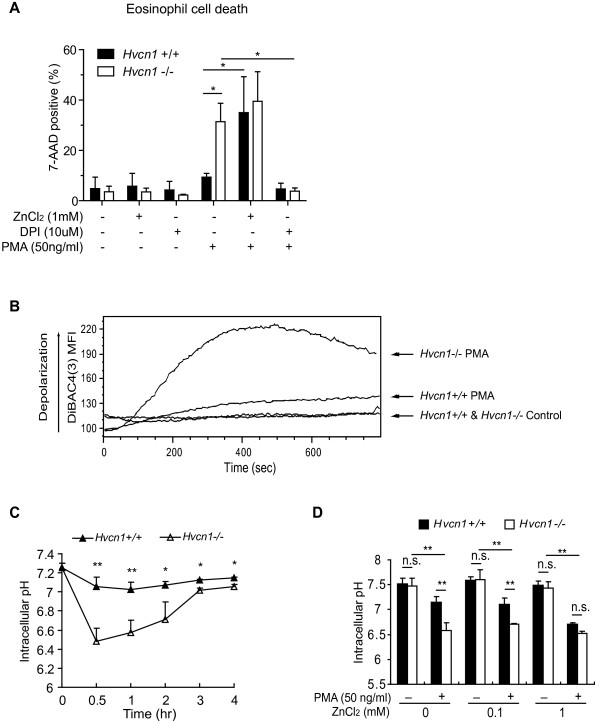
**Increased membrane depolarization and cytosolic acidification in activated HVCN1-deficient eosinophils. **(**A**) Flow cytometric analysis of eosinophil cell death (7-AAD positive cells) after four-hour PMA (50 ng/ml) stimulation in the presence or absence of ZnCl_2 _(1 mM) or DPI (10 μM). Data are expressed as the mean ± SD of 3 separate experiments. (**B**) Mean fluorescence intensity (MFI) of DiBAC4(3) in WT and HVCN1-deficient BM-derived eosinophils. PMA evoked a larger depolarization in HVCN1-deficient than WT eosinophils. The vertical arrow indicates the direction of depolarization. Data are representative of 5 separate experiments. (**C**) Intracellular pH in WT and HVCN1-deficient BM-derived eosinophils was measured using the pH-sensitive dye SNARF-4 AM (5 μM) at the indicated time points after PMA (50 ng/ml) stimulation. Dead cells were excluded by gating out cells that failed to retain SNARF-4 AM fluorescence. Data are expressed as the mean ± SD of 4 separate experiments. (**D**) Intracellular pH in WT and HVCN1-deficient BM-derived eosinophils was measured 30 minutes after PMA (50 ng/ml) stimulation in the presence or absence of ZnCl_2 _at the indicated concentrations. Data are expressed as the mean ± SD of 3 separate experiments. *, *P *< 0.05; **, *P *< 0.01; n.s., not significant.

The activation of NADPH oxidase can depolarize the plasma membrane by transporting electrons to the extracellular space [30] and simultaneously generate protons, leading to cytosolic acidification [31]. Both membrane depolarization and cytosolic acidification can cause cell death [32,33], such as that shown in Figure 4. Under physiological condition, proton efflux through the voltage-gated proton channels provides the compensating charge, thus preventing membrane depolarization and cytosolic acidification as well. Thus, in order to first test the hypothesis that PMA-induced cell death in HVCN1-deficient eosinophils was dependent on the activation of NADPH oxidase, we used the inhibitor DPI. When HVCN1-deficient eosinophils were incubated with DPI prior to PMA stimulation, cell death was prevented (Figure 5A), which suggested that PMA-induced cell death is NADPH oxidase-dependent. Second, we hypothesized that lack of HVCN1 leads to membrane depolarization and cytosolic acidification under conditions of NADPH oxidase activity. Using the potential-sensitive probe DiBAC4(3), we measured changes in membrane potential during the activation of eosinophils. As shown in Figure 5B, the membrane potential of unstimulated cells was not affected by HVCN1 expression; however, HVCN1-deficient eosinophils exhibited significantly larger increase in membrane potential (depolarization) than WT eosinophils after PMA stimulation.

Next, we performed intracellular pH measurement in eosinophils by flow cytometry with pH indicator SNARF-4 AM. The cytosolic pH of WT and HVCN1-deficient eosinophils was similar (pH = ~7.3) in the absence of PMA stimulation. However, HVCN1-deficient eosinophils had remarkable cytosolic acidification (pH = ~6.5) as early as 30 minutes after PMA stimulation. This decreased cytosolic pH was maintained for at least 2 hours (Figure 5C). In sharp contrast, WT eosinophils had only a minor decrease in cytosolic pH, which was never below pH 7.0 during the assay (Figure 5C). Notably, the addition of 1 mM Zn^2+^ induced the cytosolic acidification in PMA-stimulated WT eosinophils to yield a similar pH level as that of PMA-stimulated HVCN1-deficient eosinophils (Figure 5D). Moreover, this additional Zn^2+^ induced increased cell death in PMA-stimulated WT eosinophils similar to that in PMA-stimulated HVCN1-deficient eosinophils (Figure 5A), indicating that the inhibitory effect of Zn^2+^ on proton channel activity mimics the HVCN1 deficiency on the eosinophils. Together, these data demonstrate that proton channel HVCN1 prevents membrane depolarization and cytosolic acidification during the activation of eosinophils.

## Discussion

Recent studies have identified HVCN1 as the proton channel responsible for charge compensation during NADPH oxidase activity in neutrophils [10,12]. Additionally, unexpected roles for HVCN1 were found in neutrophils and B cells [12,13]. In this report, we used WT and HVCN1-deficient eosinophils to determine the function of HVCN1 in this cell type. Our studies revealed several novel findings. First, we demonstrate that *Hvcn1* mRNA expression is increased in allergic lung and is at a high basal level in eosinophils. Second, we show that unlike mouse neutrophils, eosinophils do not require HVCN1 for chemotaxis. Finally, we demonstrate that HVCN1 is required for prevention of activation-induced eosinophil cell death, likely because of the membrane depolarization and cytosolic acidification that occurs following PMA stimulation in the absence of HVCN1.

In the present study, HVCN1-deficient eosinophils demonstrated no migration defect, which is in contrast to the results of HVCN1-deficient neutrophils. This can be due to the different mechanism involving calcium mobilization which controls numerous cellular functions including cell migration. In neutrophils, Ca^2+^ entry occurs largely across store-operated Ca^2+^ channels from the extracellular environment [34]. Thus, the increased membrane depolarization can reduce the driving force for extracellular Ca^2+^ into the cells and as a result impair migration ability, which has been shown in fMIVIL-activated HVCN1-deficient neutrophils [12]. However, in human eosinophils, several studies showed that pre-incubation of eosinophils by intracellular calcium chelator 1,2-bis-(*o*-aminophenoxy) ethane-*N,N,N,N*-tetraacetic acid acetoxy-methyl ester (BAPTA-AM) dose-dependently prevented calcium flux and the chemotactic response to platelet activating factor (PAF) and complement fragment 5a (C5a), but the depletion of extracellular calcium had no effect [35,36], suggesting that intracellular calcium plays a more important role in regulating eosinophil migration. The other potential explanation (not mutually exclusive) for the lack of effect of HVCN1 deficiency on eosinophil migration comes from the finding that mEotaxin-1 does not induce significant ROS generation by mouse eosinophils [37], which suggests that mEotaxin-1 does not activate the NADPH oxidase and thus induce the membrane depolarization in HVCN1-deficient eosinophils. Together, HVCN1 deficiency does not affect mouse eosinophil migration.

In our study, we found that HVCN1 was required for optimal ROS production. However, HVCN1-deficient eosinophils still had ~50% ROS production retained (Figure 2A-D). Similarly, HVCN1-deficient neutrophils and B cells had ~30% ROS production retained [10,13], suggesting that proton channels are not indispensible even though they provide the bulk of compensating charge in phagocytes [38] and that other channels might facilitate charge compensation during ROS generation. For instance, ClC-3 Cl^-^/H^+^ antiporter [39,40], CLIC-1 Cl channels [41], TRPV1 nonselective cation channels [42], SK2 and SK4 Ca^2+^-activated K^+^ channels [43], and Kv1.3 delayed rectifier K^+^ channels [44] have been proposed to contribute to charge compensation in leukocytes. However, while these channels might provide charge compensation, they would not alleviate the acidification. In contrast, the sodium-hydrogen exchanger could extrude the extra acid but could not compensate the extra charge as this exchanger is not electrogenic [45,46]. Thus, HVCN1 is ideally suited to facilitate charge compensation during ROS generation as it provides both charge and acidity compensation.

HVCN1 was required for optimal ROS production by eosinophils. Our study suggests two likely reasons for this finding: (1) HVCN1-deficient eosinophils were more depolarized than WT eosinophils after PMA stimulation (Figure 5B), which was most likely caused by the lack of compensating charge provided by proton channels. As a result, the membrane depolarization hinders the flow of electrons across the voltage-dependent flavocytochrome [5,6], which is needed to reduce oxygen to superoxide. (2) The deficiency of HVCN1 was also associated with a substantial cytosolic acidification, which happened as quickly as within 30 minutes after PMA stimulation (Figure 5C). The activity of NADPH oxidase responsible for ROS production, being optimal at intracellular pH 7.0-7.5, could be decreased in this resulting acidic cytosol [47].

In addition to inhibiting NADPH oxidase activity, membrane depolarization and cytosolic acidification may be responsible for the observed activation-induced cell death. However, we cannot exclude other possible reasons. For instance, imbalance of osmolarity across the membrane (presumably caused by K^+^ efflux) might also account for the increased cell death of HVCN1-deficient eosinophils following PMA stimulation.

In summary, our study identifies cell-specific roles for HVCN1 in eosinophil respiratory burst and prevention of activation-induced cell death but not eosinophil migration. These findings have implications for our understanding of the basic mechanism of eosinophil function, as well as for targeting of eosinophils in eosinophil-associated diseases.

## Conclusion

Eosinophils require proton channel HVCN1 for optimal ROS generation and prevention of activation-induced cell death.

## Abbreviations

BALF: Bronchoalveolar lavage fluid; BM: Bone marrow; DHR 123: Dihydrorhodamine 123; DiBAC4(3): Bis-(1,3-dibutylbarbituric acid) trimethine oxonol; DIDS: 4,4′-Diisothiocyanatostilbene-2,2′-disulfonic acid disodium salt hydrate; DPI: Diphenylene iodonium; HRP: Horseradish peroxidase; NFA: Niflumic acid; NPPB: 5-Nitro-2-(3-phenylpropylamino)benzoic acid; PMA: Phorbol myristate acetate; ROS: Reactive oxygen species; SCF: Stem cell factor; SOD: Superoxide dismutase; WT: Wild-type.

## Competing interests

The authors declare that they have no competing interests.

## Authors’ contributions

XZ participated in study design, performed experiments, data analysis and manuscript writing; EM performed experiments; NZ supervised study design, data analysis and manuscript writing. All authors read and approved the final manuscript.
